# Description of an Annular Saw

**Published:** 1815-03

**Authors:** 

**Affiliations:** Surgeon, of Wolsingham.


*For the Medical and Physical Journal.*

### *Description of an Annular Saw;* by Mr. MACHELL, Surgeon, of Wolsingham.

WHEN an invention is novel, in both application and principle, it is somewhat difficult to convey a clear and correct idea, by any drawing or description, that may be generally understood; but I am induced to give the following description of my Annular Saw, from the very flattering reception it has met with from the Earl of Stanhope, by whose approval and patronage I have been honoured, and also by several surgeons of the highest professional eminence in the kingdom, by whom the Annular Saw is acknowledged to supersede the use of the trephine, trepan, Hey's saws, bone nippers, rasp, the mallet and chissel, &c. &c. in all cases where any portion of carious bone demands the interference of our art to remove it. These instruments which I have recited above, are the best at present in use, in a variety of surgical cases where the common saw cannot possibly be applied; and there are also many cases where even these instruments cannot be used but with the greatest regard to the patient; as in the cure of simple fractures, where there is a want of union, by the bone ends rubbing against each other, from which they become smooth, and are thereby prevented from uniting. We are advised by authors, to lay bare the fractured ends of the bones, and to remove a portion of their callous and smooth edges. This operation has been repeatedly performed, yet I believe with little success, which may be owing to the great extent of muscular surface unavoidably exposed, together with the injury inflicted upon other important parts, in order to apply such instruments that have been hitherto used in performing the operation. The Annular Saw is considered as well adapted for removing a piece of bone in similar cases with celerity and safety, without injuring any of the large blood-vessels and nerves that may lie contiguous or immediately in the neighbourhood of the fractured part; *here* the incision must be made, and *need* only be of length sufficient to admit of the soft parts being retracted to the width of about an inch, a little more than the diameter of the bone, for the purpose of applying the saw, and which should be, as near as as we are able to judge, equal to the bone's diameter. The great length of time so formidable an operation requires in the ordinary mode of proceeding, is, to most surgeons, of little practical importance, as the success of cure is thought to be no way retarded; yet, when the length of time we are engaged in performing an operation is calculated by *minutes* instead of *hours*, this will undoubtedly be a desideratum to the patient.

Perhaps the following case will demonstrate its decided advantages over any other surgical instrument at present in use, and from this, its application in many similar cases will be at once understood.

### *A Case of Necrosis.*

In 1806, I had under my care, J. Taylor, a young man who resided at Witton-le-Wear, county of Durham, and who had become very weak and emaciated from a disease of fourteen years' duration, and had dragged about one of the worst sort of ulcerated legs. At the time I first saw him, he had night perspirations, alarming hectic, and great prostration of strength. On examining the limb, I found it a complete case of Necrosis, and determined to remove, if possible, the sequestra, which was inclosed by a case of new bone: this was also perforated by a number of holes of the size of a goose-quill, and from which a copious sanious discharge was pouring out daily. Neither the head of the trephine nor Hey's saw could be admitted, on account of the great destruction to the surrounding soft parts that they would necessarily occasion; recourse was therefore had to the mallet and chissel, but, on the slightest attempt, the pain was so acute, and he, having previously refused amputation, now chose rather to die than submit to the operation being completed; so that for want of other proper means to remove the carious bone, it was at this time deferred without much hope of ever being able to relieve him. Horrid as these instruments already described may appear, they are the best at present in use in such cases, and are strenuously recommended by our first surgeons, and particularly by Mons. Bousslin. However, after a short absence from my patient, an idea occurred to me, that an instrument might be constructed which would be of considerable advantage in this case, and I delayed no time in communicating it to him, or in getting my design accomplished. When I again saw him, I was prepared to begin the operation, being then under promise to my patient to remove the diseased bone, without making use of such blows as he had before experienced from the mallet and chissel, and which, with those instruments, could not possibly sibly be avoided. I first cleared away for the space of an inch and an half by about an inch, the integuments and periosteum, which had become much thickened, down to the bone at the upper part of the leg, when the new bone, to the dimensions described above, was very easily sawed through with the Annular Saw, which exposed the upper end of the sequestra: this part was cut off transversely, again divided longitudinally, and then extracted in two pieces; the remaining part was laid hold of by the common forceps, drawn up to the top of the opening, and then sawed off: this was repeated thrice, and the whole sequestra, being seven or eight inches in length, was sawed into pieces in the middle of the leg, and extracted by an opening in the integuments and new bone of the above dimensions. The violent irritation which the diseased bone had occasioned, obliged him to have constant recourse to opium with good effect, and in the space of six months, he was so far recovered as to be able to walk without a stick, and was restored to perfect health.

Dr. Clanny, a most respectable physician of Sunderland, was present during the operation, and afterwards expressed his opinion in the following letter:

"I witnessed the operation in which you used your Annular Saw, in the case of Necrosis, at Witton-le-Wear, in the summer of 1806, with much pleasure and satisfaction. In my opinion, a more ingenious instrument for the purposes which you designed it could not be devised, and have no doubt but you will be amply rewarded by a discerning public. W. Reed Clanny, M.D.

"*To Mr. Thomas Machell, Surgeon, Wolsingham.*"

The three following certificates are from surgeons of the highest professional character:—

"*London, Sackville-street, June* 15, 1812.

"Mr. Machell has shown me a circular saw, which he has applied to the purposes of sawing bones. It is a very ingenious invention, and applicable to many cases of surgery: he is, therefore, highly deserving of encouragement. Everard Home."

"I have examined Machell's Annular Saw, at the request of the inventor (Mr. T. Machell), and it appears to me to be exceedingly well calculated, from its mode of operation, to divide bones, or protrusions of splintered bones; consequently very frequently, or more immediately, to gun-shot wounds; and for which I know of no instrument in use capable of being equally applicable in such cases, and it is deserving of protection. J. Charlton,

"Surgeon-Major, 1st Foot Guards.

"*London, 5th June,* 1812.

"*To Mr. Thomas Machell, Surgeon, Wolsingham.*"

 "I think

"I think the Annular Saw, invented by Mr. Machell, will be found very useful for the operations in surgery in which he proposes to use it. "J. DORATT.

"*London, Bruton-street,*
*June* 3, 1812."

Mr. Ingham, a surgeon of the first eminence in Newcastle, has also favoured me with his opinion of the Annular Saw:—

"Sir,—It appears to me that your improved Saw is well contrived to saw bone in various surgical operations with expedition and facility. I am, Sir, your's, faithfully,

"WM. INGHAM.

"*Newcastle, April* 18, 1814.

"*To Mr. Machell, Surgeon, Wolsingham.*"

---

*For the Medical and Physical Journal.*

